# A concept for optimizing avalanche rescue strategies using a Monte Carlo simulation approach

**DOI:** 10.1371/journal.pone.0175877

**Published:** 2017-05-03

**Authors:** Ingrid Reiweger, Manuel Genswein, Peter Paal, Jürg Schweizer

**Affiliations:** 1 BOKU University of Natural Resources and Life Sciences, Department of Civil Engineering and Natural Hazards, Vienna, Austria; 2 Independent Researcher, Meilen, Switzerland; 3 Department of Perioperative Medicine, Barts Heart Centre, William Harvey Research Institute, Barts & The London School of Medicine&Dentistry, Queen Mary University of London, London, United Kingdom; 4 Department of Anaesthesiology and Intensive Care Medicine, Hospitallers Brothers Hospital, Salzburg. Teaching Hospital, Paracelsus Medical University, Salzburg, Austria; 5 WSL Institute for Snow and Avalanche Research SLF, Davos, Switzerland; Azienda Ospedaliero Universitaria Careggi, ITALY

## Abstract

Recent technical and strategical developments have increased the survival chances for avalanche victims. Still hundreds of people, primarily recreationists, get caught and buried by snow avalanches every year. About 100 die each year in the European Alps–and many more worldwide. Refining concepts for avalanche rescue means to optimize the procedures such that the survival chances are maximized in order to save the greatest possible number of lives. Avalanche rescue includes several parameters related to terrain, natural hazards, the people affected by the event, the rescuers, and the applied search and rescue equipment. The numerous parameters and their complex interaction make it unrealistic for a rescuer to take, in the urgency of the situation, the best possible decisions without clearly structured, easily applicable decision support systems. In order to analyse which measures lead to the best possible survival outcome in the complex environment of an avalanche accident, we present a numerical approach, namely a Monte Carlo simulation. We demonstrate the application of Monte Carlo simulations for two typical, yet tricky questions in avalanche rescue: (1) calculating how deep one should probe in the first passage of a probe line depending on search area, and (2) determining for how long resuscitation should be performed on a specific patient while others are still buried. In both cases, we demonstrate that optimized strategies can be calculated with the Monte Carlo method, provided that the necessary input data are available. Our Monte Carlo simulations also suggest that with a strict focus on the "greatest good for the greatest number", today's rescue strategies can be further optimized in the best interest of patients involved in an avalanche accident.

## Introduction

In many countries the total number of avalanche victims did not increase during the last decade (e.g., [1]) despite a growing number of recreationists in snow-covered, mountainous terrain. However, in some countries such as Canada, France, Norway, and Switzerland, the number of avalanche incidents with multiple victims has increased in recent years. In Switzerland, for instance, the number of people involved in fatal avalanche accidents has increased by almost 50% in the five winters from 2008–2009 to 2012–2013 compared to the preceding five winters. At the same time the number of accidents with three or more fatalities has doubled as well [2]. This suggests that avalanche accidents where several persons require rescue and medical assistance are increasing. The first 35 min of an avalanche accident are most critical because the completely buried subjects with an obstructed airway suffocate quickly. At the same time rescue resources are often limited, making it impossible to provide the best standard care to all extricated and still buried subjects simultaneously. Strategies which help to allocate the limited resources to the most likely lifesaving tasks (i.e. triage strategies), are required [3].

Apart from technical developments, e.g. improving the performance and user friendliness of search devices, it seems most promising to improve rescue efficiency by optimizing rescue procedures with a strict focus towards saving as many lives as possible, a concept which is also known as the “greatest good for the greatest number” theorem (Jeremy Bentham, 1748-1832).

Based on the “greatest good for the greatest number” consideration Bogle *et al*. [4] developed the Avalanche Survival Optimizing Rescue Triage (AvSORT) algorithm. They made theoretical considerations based on medical data and estimated survival probabilities of avalanche victims under different circumstances. Based on their considerations they state that “it would be for the greater benefit of the whole to extract potential survivors that risk asphyxia while buried rather than engage in protracted resuscitation on probable non-survivors”. This is exactly the point we will focus on in the second example we present below, not only based on estimations but also with probabilistic calculations applying the Monte Carlo method using real data.

A first technical attempt to apply numerical simulations based on the Monte Carlo principle on an avalanche accident scenario was presented by Genswein *et al*. [5]. They optimized the search strip width for signal search with an avalanche transceiver, i.e. the width between two search strips when searching an avalanche deposit larger than the effective range of the transceiver. Depending on the avalanche deposit size the resulting “survival chance optimized” search strip widths were clearly larger than some of the common recommendations–exemplifying the potential for improving avalanche safety by optimized rescue strategies.

As in the case of the search strip width, there is usually a trade-off between competing processes involving complimentary parameters, such as e.g. speed and accuracy. If the probability distributions between both the first parameter (e.g. speed) and survival chance as well as the second parameter (e.g. accuracy) and survival chance are known, it is possible to calculate the optimal parameter setting in order to maximize the survival chances with respect to the “greatest good for the greatest number”.

If the probability distributions are known only empirically, as it is the case for distributions stemming from the relatively small database of avalanche accidents (e.g. the distribution for avalanche burial depth, or avalanche deposit size), performing a Monte Carlo simulation [6] to calculate a parameter setting maximizing survival chances seems most promising.

The Monte Carlo (MC) method was originally developed by Metropolis *et al*. [6], who described it as a "general method, suitable for fast electronic computing machines, of calculating the properties of any substance which may be considered as composed of interacting individual molecules". Since the 1950s when the MC method was introduced, the development of "fast electronic computing machines" has advanced enormously, and Monte Carlo simulations have become a standard tool for performing numerical simulations including stochastic or probabilistic variables (e.g. [7]).

In this study, we aim to demonstrate how Monte Carlo simulations can be applied to optimize avalanche rescue strategies. We do so for two exemplary rescue situations where competing, incompatible requirements exist. In a first practical example, we calculate an optimal probing depth in the first passage of a probe line search for one buried subject. Here, there is a trade-off between the chances of finding the buried subject (accuracy) and the time it takes for finding the buried subject (speed), i.e. the burial duration, which strongly influences the probability of survival. As a second application, we used a Monte Carlo simulation for demonstrating how to calculate the optimal duration of resuscitation performed by a single rescuer on a not-breathing patient, i.e. already excavated patient, in case of another buried subject not yet excavated. The scenario of one rescuer and two buried subjects both needing urgent rescue and medical assistance is the simplest case of shortage of resources–a setting requiring a triage criterion–within avalanche rescue.

## Methods

The Monte Carlo method is a numerical method to solve mathematical problems by random sampling [7]. We used the method to calculate expectation values of quantities depending on random variables whose values were taken from an empirical probability distribution. Our Monte Carlo simulation was based on repeated random sampling, i.e. randomly taking parameter values according to their empirical probability distribution and calculating expectation values of interest with the sampled parameters. Monte Carlo methods are generally of particular usefulness and importance in cases where the calculation of the expectation value is not possible analytically. For the variables, which are of interest within avalanche rescue the existing distributions are empirical data sets for which we mostly do not have analytical formulas.

In simplified form the Monte Carlo method consists of the steps listed below. As an example, we include the specifics steps for the probing depth simulation. Note that we use bold italic letters for random variables and quantities depending on random variables to distinguish them from all other variables or parameters (for which italic letters are used).

- Define the distributions, either empirical or analytical, of the random variables of interest. In the considered example random variables are: i) the lateral position of the buried subjects: uniform distribution and ii) the burial depth: empirical data from the SLF avalanche data base.
- Generate a large number of values for the random variables, each value is chosen randomly according to its probability distribution. In our example, by using a random number generator, we generate uniformly distributed random numbers i) from position 1 to position “end”–this position is used for placing the buried subject laterally and ii) from 1 to the maximum number of data points, we then take the value of this data point as our burial depth.
- Perform deterministic calculations of the quantity of interest, using the randomly chosen set of values for the random variables as input. Each calculation with a different set of values represents a simulation run. In our probing depth example, we calculate the survival probability using randomly chosen lateral position and burial depth from above.
- The mean value for the quantity of interest can be estimated by repeating the simulation a predefined number of times, thus generating the distribution for the quantity of interest from which respective values such as mean or variance can be calculated. In our example this means, calculating the mean survival probability from the results of all simulation runs for a given probing depth.

We applied the Monte Carlo method in two scenarios within avalanche rescue–probe line search and resuscitation strategy in case of an additional buried subject–that are described in detail below.

### Optimal probing depth for maximizing survival chances

A probe line search is a search method, which is applied when buried subjects cannot be located by electronic search means (e.g. transceivers or Recco) or an avalanche dog. It involves penetrating the avalanche debris with long probes [8] until a distinct difference in tactile feedback (e.g. elasticity, hardness) indicates that the tip of the probe has made physical contact with a human body. For this purpose, rescuers are trained to be able to distinct a human body from snow, the underlying terrain, or solid material like rocks, ice, and branches of trees.

As the survival chance of a buried subject decreases dramatically with increasing burial time [9, 10], the success of a probe line search is not only determined by accuracy (probability of detection) but also by speed. Increasing the probing depth–i.e. penetrating the probe deeper into the avalanche debris–increases the chances of finding a deeply buried subject, but reduces search speed. A shallow probing depth on the other hand, favours the survival chances of a victim not buried deeply but at the far end of the avalanche debris.

The strategy of limiting probing depth to improve the odds of finding those victims that are less deeply buried more quickly is not new [11]. More recently, Ballard *et al*. [12] presented a computer program to simulate a fully articulated human body buried in an avalanche debris, and then implement a certain probing technique. The program compares the probability of detections for different grid patterns of probing. Within the present work we assume the slalom probing technique presented by Genswein *et al*. [13].

Following the approach of the “greatest good for the greatest number” we performed a Monte Carlo simulation to find an optimal probing depth for maximizing the survival chance of a buried subject. We focused on the Monte Carlo method for determining an optimal probing depth, other important considerations such as the search strategy are described by Genswein *et al*. [13] who also provide a detailed description of avalanche probing and the effect of spacing the probing strokes on the victim’s survival chances.

**Fig 1:**
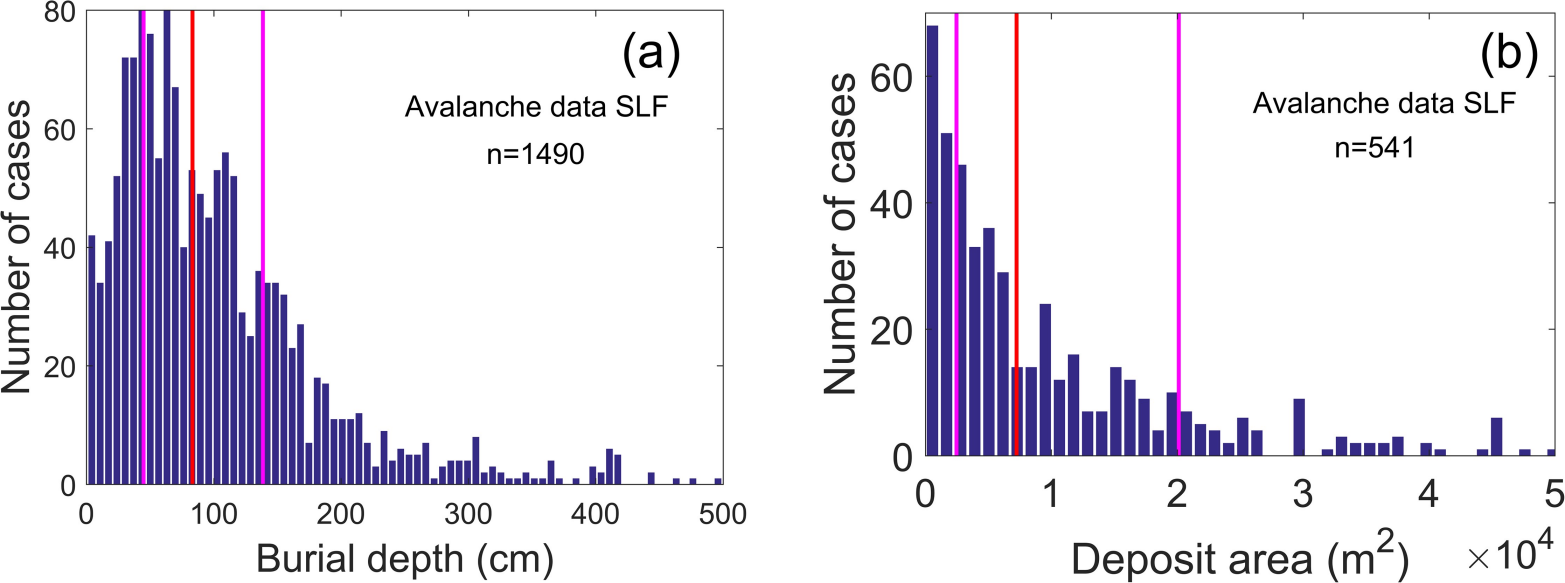
Burial depth and avalanche deposit area. (a) Burial depth for 1490 buried subjects, and (b) avalanche deposit area for 541 avalanches. The vertical lines mark the first, second (median, red line), and third quartile. Both data sets are from SLF’s avalanche data base.

The Monte Carlo simulation for calculating an optimal probing depth was performed as follows. We drew random values for both the position of the buried subject–using a uniform distribution–and its burial depth–using the avalanche database. This was repeated for six different areas of avalanche debris (50, 100, 500, 1000, 5000 and 10,000 m^2^; Fig 1). While the subject’s lateral position was sampled from a uniform distribution, the burial depth was taken from the data base compiled by the WSL Institute for Snow and Avalanche Research SLF. The data set includes the burial depth of the fully-buried subjects caught in an avalanche accident in Switzerland between 1973–1974 to 2012–2013, 1490 cases in total, see S1 Metadata and S1 Table.

This sampling procedure was repeated 10,000 times for each size of avalanche debris and 21 equally spaced probing depths ranging from 0.5 to 2.5 m. The convergence of the simulation was checked empirically, and 10,000 simulation runs appeared sufficient. Each simulation run proceeds as described below.

**Fig 2:**
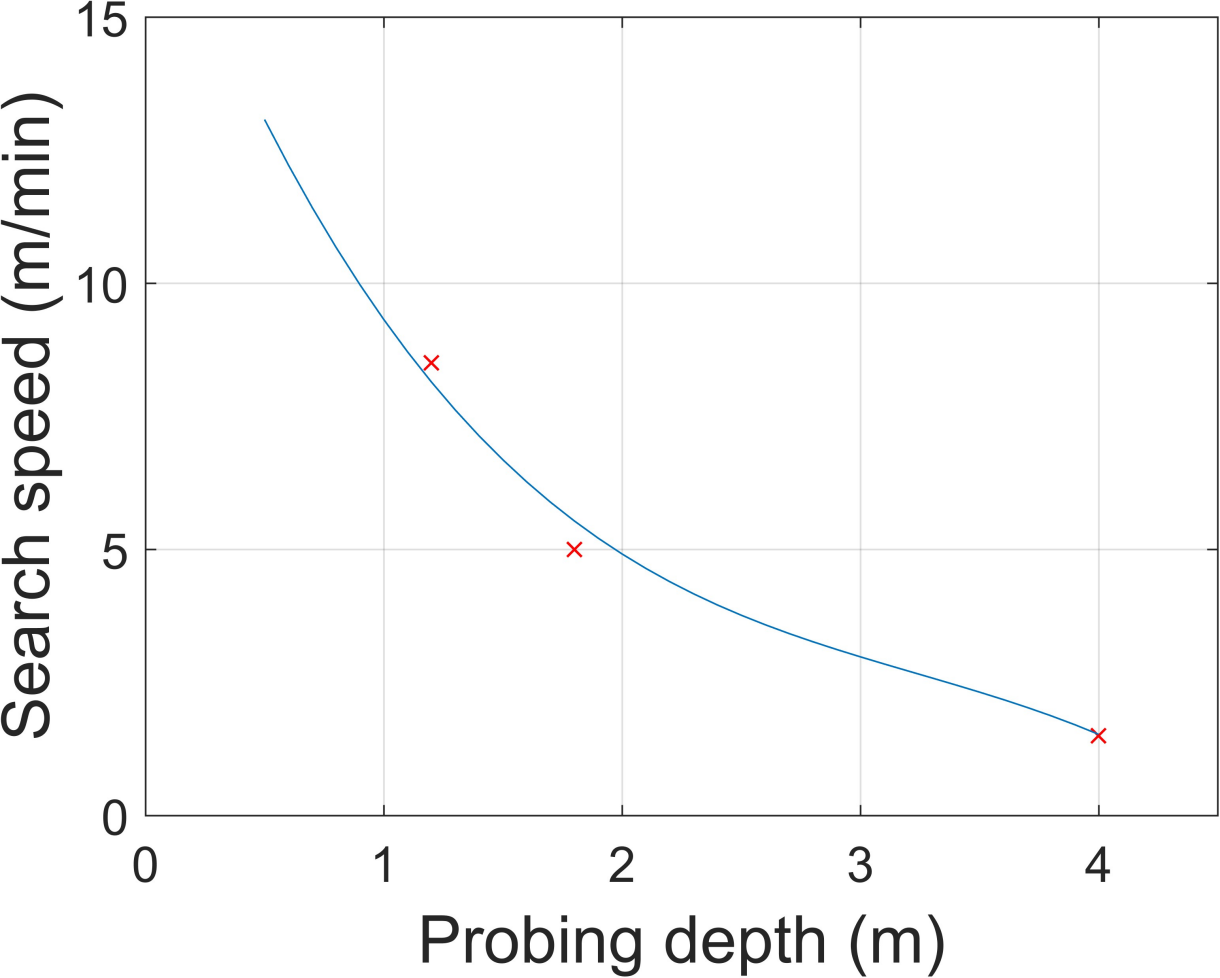
Search speed. The length (in meters) of search area (width 1.5 m) on avalanche debris covered by one probing rescuer per minute as a function of probing depth using the slalom probing technique [13].

**Fig 3:**
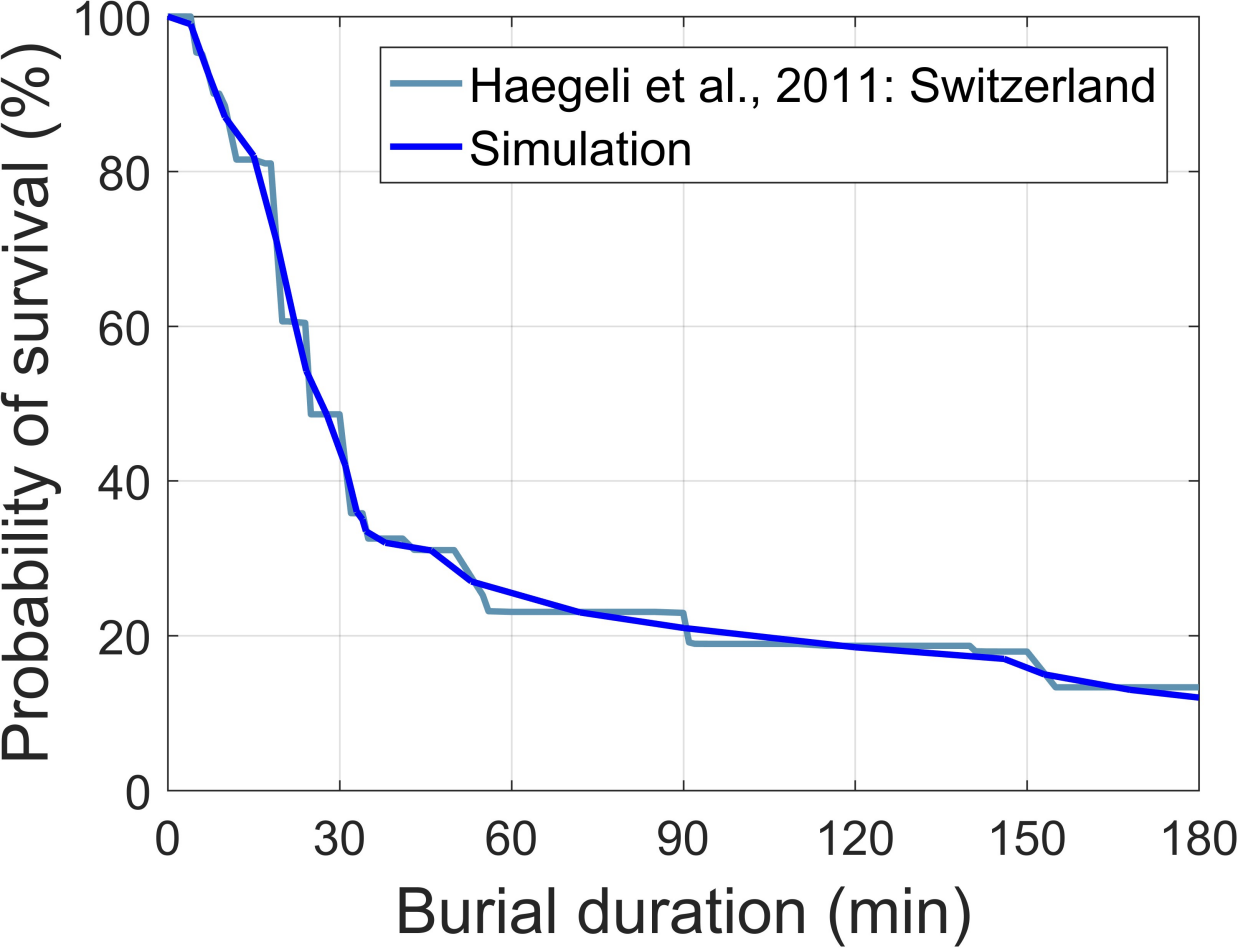
Survival chances of an avalanche burial as a function of burial time. For the simulation a smooth interpolation (blue line) of the avalanche survival curve based on Swiss accident data was used, adapted from [10].

- The randomly sampled burial depth was compared to the probing depth. If the victim was buried deeper than the probing depth, the victim was missed and the probability of survival set to zero.
- If the probing depth was larger than the burial depth, the victim was found. We did not consider lateral misses during probing. The total burial time was the sum of search and excavation time. The search time was ***t***_search_ = ***x*** / (*v*_search_ *n*_rescuers_), and the excavation time was ***t***_dig_ = ***d***_burial_ / *v*_dig_. The variables denote time (*t*), position (***x***), speed (*v*), and depth (***d***). Within the probing simulation for deposit sizes of 500 and 1000 m^2^, we used a fixed number of rescuers, *n*_rescuers_ = 5. The scenarios for deposits sized between 5000 and 100,000 m^2^ were also calculated but assuming that 20 rescuers were available to probe. We assumed the slalom probing strategy [13] with a search speed between 13 and 1.5 m per minute and rescuer depending on probing depth (Fig 2). The rescuers were supposed to use the V-shaped conveyer belt method for digging [14], the digging speed was assumed 0.15 m per minute, accordingly.
- The total burial time was calculated as ***t***_search_ + ***t***_dig_. Given the burial time the probability of survival was calculated. For the probability of survival ***p***_survival_ we used a smooth interpolation of the survival curve based on the Swiss avalanche survival data presented by Haegeli *et al*. [10] (Fig 3).

Finally, the average probability of survival for a given probing depth was calculated as the average over the survival probabilities of each of the 10,000 simulation runs.

### Resuscitation strategy in the case of an additional buried subject

Our test scenario is simple, but demands for a triage decision due to shortage of resources. We assume two buried subjects, but only one rescuer. The simulation starts as soon as the rescuer has excavated the first buried subject. The excavated patient, from now on referred to as patient 1, has no obvious lethal injuries, is normothermic, but has no vital signs. The second buried subject, referred to as patient 2, is still buried. We assume that the rescuer has witnessed the avalanche, i.e. the duration of burial is known; it is assumed an upper estimate of the time when the (apparent) cardiac arrest of patient 1 has happened.

The goal of every rescue is to save as many lives as possible. As only one rescuer is available, this person can either engage on resuscitation of patient 1, or excavate patient 2. The situation demonstrates that the shortage of resources leads to the tricky situation as we have two competing processes: the increase of survival chance of patient 1 as a function of resuscitation duration [15], and the decrease of survival chances due to increasing burial time for patient 2 [10].

In the above situation, the avalanche resuscitation algorithm as defined by the European Resuscitation Council [16] suggests to perform cardiopulmonary resuscitation (CPR) on patient 1 either until sustained return of spontaneous circulation or for a maximum of at least 20 min before proceeding to patient 2. The triage algorithm AvSORT presented by Bogle *et al*. [4], on the other hand, recommends not to spend any time on patient 1 who is not breathing despite unobstructed airways, but to continue searching for and excavating patient 2 –who is a potential survivor–immediately.

Performing CPR for a maximum of at least 20 min strongly favours the survival chances of patient 1 (*p*_1_) at the cost of the survival chances (***p***_2_) of patient 2. Proceeding to patient 2 immediately, on the other hand, while optimal for patient 2, reduces the survival chances of patient 1.

We performed a Monte Carlo simulation to calculate the optimal time for performing CPR on patient 1 before proceeding to patient 2 in order to maximize the expected number of survivors. The number of expected survivors for *n*_1_ and *n*_2_ persons with respective survival probabilities *p*_1_ and *p*_2_ is calculated as *n*_1_∙*p*_1_+ *n*_2_∙***p***_2._ Since we have *n*_1_ = *n*_2_ = 1, the expected number of survivors simplifies to *p*_1_ + ***p***_2_. The probability, that both patients survive can be calculated as *p*_1_∙***p***_2_, and the probability that at least one of them survives is given by 1 − [(1 − *p*_1_) ∙ (1 − ***p***_2_)]. These calculations are valid since *p*_1_ and ***p***_2_ are independent, i.e. do not intrinsically depend on each other.

We tested different times for performing CPR on patient 1 (*t*_CPR_) and calculated the average number of survivors for each *t*_CPR_, ranging from 0 to 30 min in one minute steps. Each simulation consisted of 10,000 runs. We empirically checked the simulation's convergence by varying the number of simulation runs. Once the simulation results did not change anymore with increasing number of runs, we considered the simulation as converged. The simulation steps of our Monte Carlo simulation are described in detail below.

**Fig 4:**
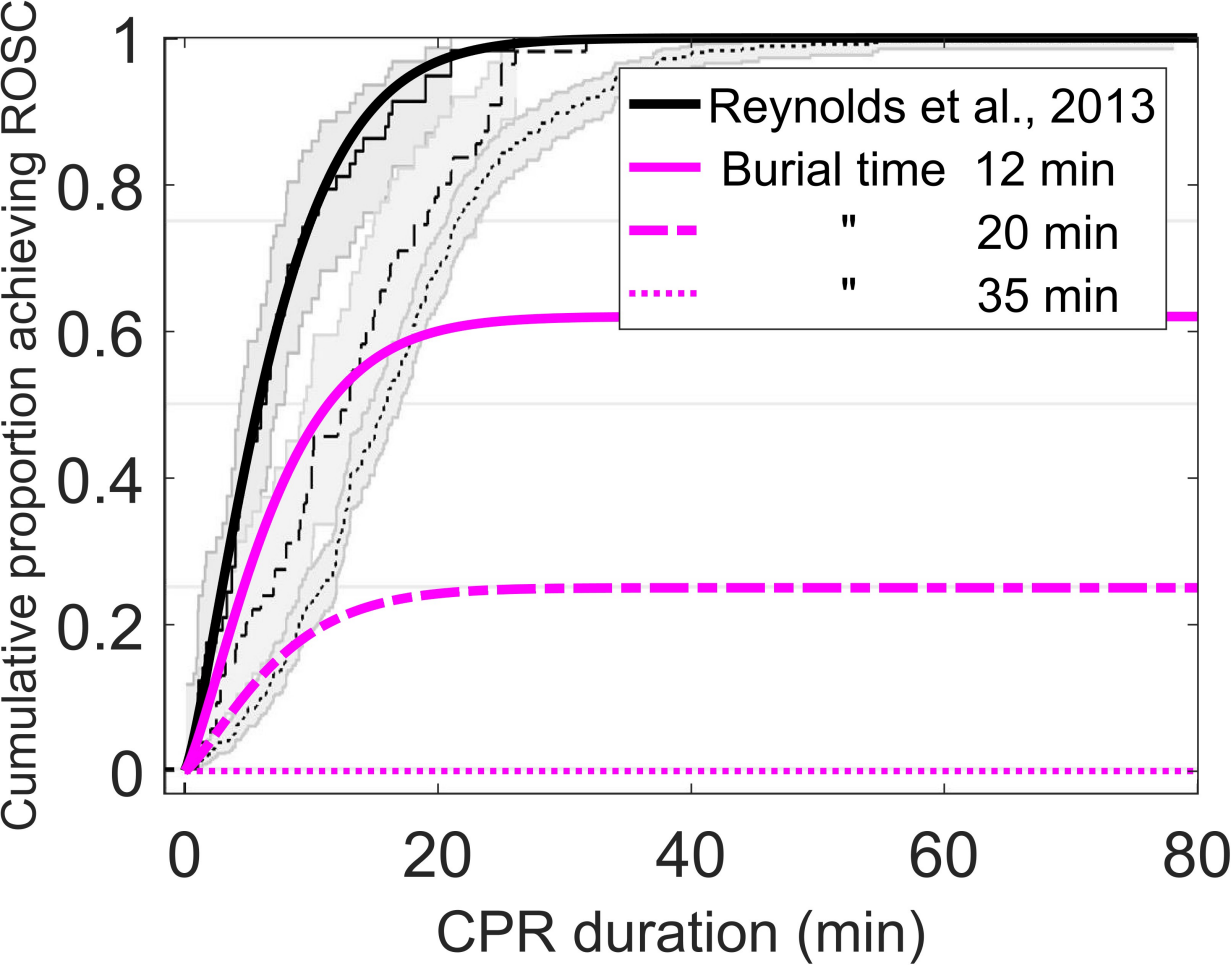
Probability of achieving return of spontaneous circulation (ROSC) depending on the duration of the cardiopulmonary resuscitation (CPR). The magenta curves refer to the three scenarios of burial time for patient 1, namely 12, 20, and 35 min; adapted from Reynolds *et al*. [15]. The maxima of the magenta curves are calculated according to the data from Moroder *et al*. [18].

1. The set of random variables and parameters we need for each simulation run were defined as follows:
  - Search time ***t***_search_ for patient 2. This parameter was drawn from a Gaussian distribution with mean of 2 min [17] and a standard deviation of 1 min. Negative values were set to zero.
  - Excavation time ***t***_dig_ for patient 2. The excavation time depends on the burial depth. We used the data-based burial depth distribution shown in Fig 1 and a digging speed of 0.15 m per minute [14].
2. We generated a large number of values for the random variables ***t***_dig_ and ***t***_search_ drawn from the respective probability distributions. Note that ***t***_dig_ and ***t***_search_ are assumed to be independent.
3. We deterministically calculate the expected number of survivors (*p*_1_+***p***_2_) with each set of values for the random variables. The survival probability of patient 1, *p*_1_, is estimated based on data presented in Fig 4 [15]. The curves in Fig 4 can be approximated by *p*_1_ = *a* (1 - exp [-0.07 *t*_CPR_^1.3^]) with *a* describing the portion of subjects who survive. While the shape of the curves was taken from the data of Reynolds *et al*. [15], the values of the parameter *a = f* (*t*_burial, patient1_) as a function of the initial burial time of patient 1 as well as the chosen values for the initial burial time are based on the data presented by Moroder *et al*. [18]. The survival probability of patient 2, ***p***_2_, is calculated based on the curve in Fig 3 [10]. The duration of burial of patient 2 is the sum of *t*_burial, patient1_ *+ t*_CPR_ + ***t***_search_ + ***d***_burial_ / *v*_dig_. The time *t*_CPR_ was a fixed parameter ranging from 0 to 30 min.
4. We calculated the average expected number of survivors (*p*_1_+***p***_2_) over all simulation runs.

A simulation as described above was done for each *t*_CPR_ ranging from 0 to 30 min and four different assumed burial times for patient 1, namely 12, 20, and 35 min, and corresponding values of *a* = 0.62, 0.25, 0, respectively. The values for *a* were calculated from the data presented by Moroder *et al*. [18].

## Results and discussion

### Optimal probing depth

**Fig 5:**
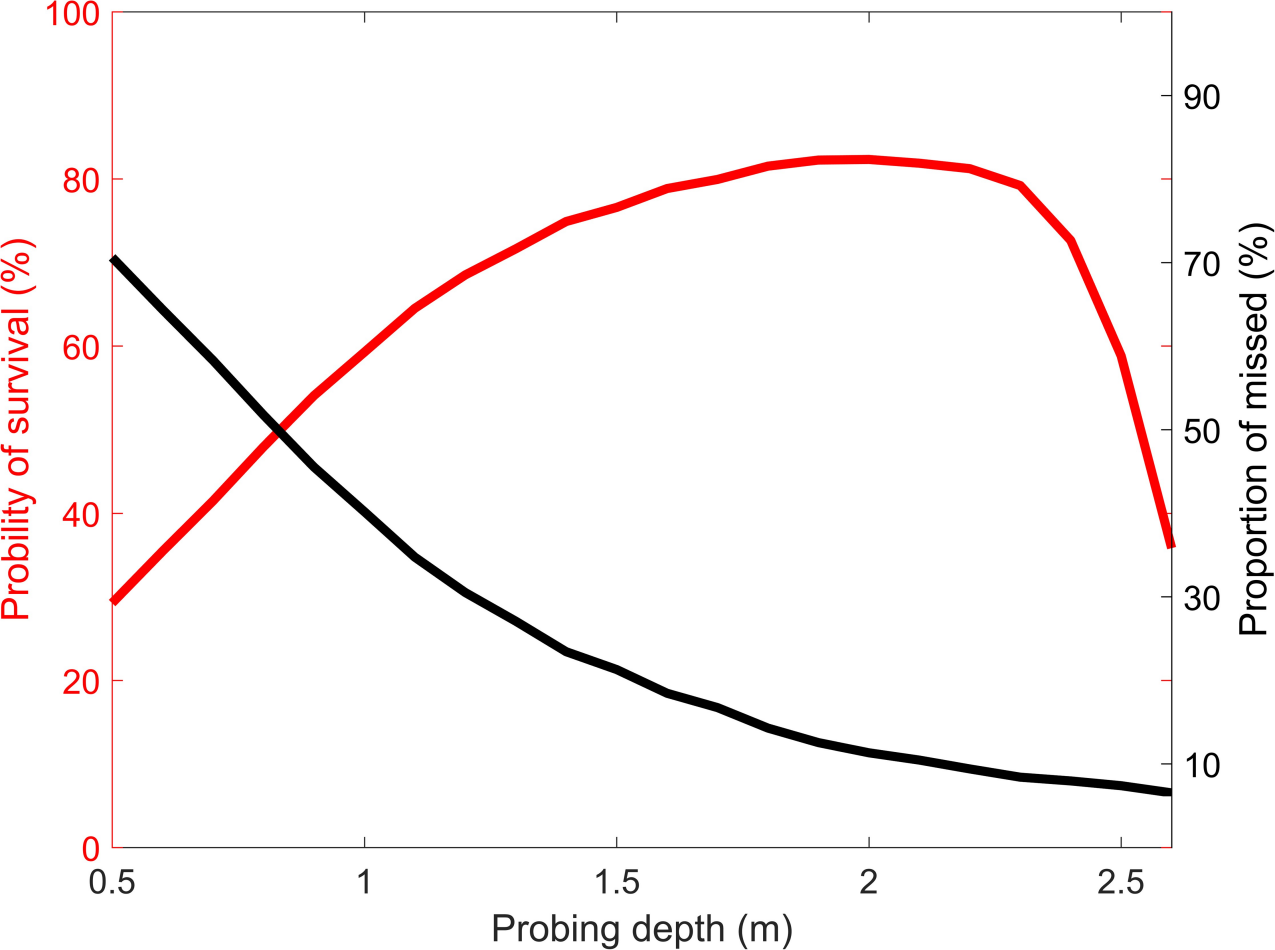
Probability of survival. Probability of survival (red curve) of an avalanche victim as a function of different probing depths for a search area (avalanche debris size) of 5000 m^2^. Also shown (black curve) is the probability of missing the buried subject.

An exemplary result for a debris size of 5000 m^2^ is shown in Fig 5. It can be seen that for a shallow probing depth of 0.5 m roughly 70% of the avalanche victims were missed. As the number of misses decreased with increasing probing depth, the probability of survival ***p***_survival_ initially increased. At a probing depth of 1.9 m, however, the probability of survival reached its maximum before decreasing again. This decrease of ***p***_survival_ at large probing depths was due to the decrease of the victim’s survival probability with longer burial time (Fig 3). The decrease in survival probability with longer burial duration implies that while more than 90% of all victims were found with a large probing depth of 2.5 m, many of them already died when they were finally dug out, since they had been buried in the snow for too long, i.e. the search was too slow. So there was an optimal trade-off between accuracy (large probing depth) and speed (shallow probing depth). An optimal value in the sense of maximizing the victim’s survival chances was a probing depth of roughly 2 m for a search area of 5000 m^2^.

**Fig 6:**
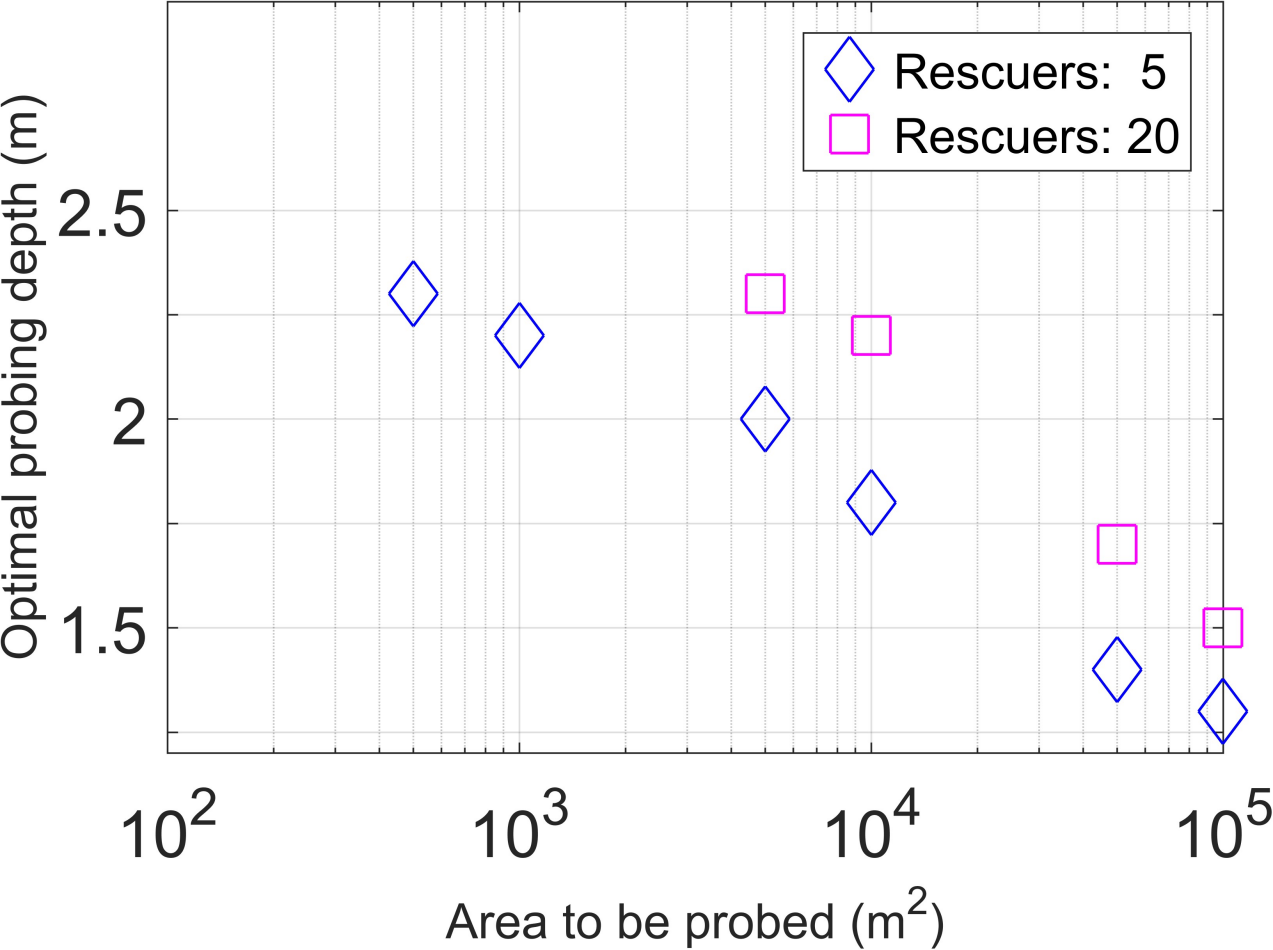
Optimal probing depth. Probing depth leading to the highest survival chance for a buried subject as a function of area to be probed for either five (blue diamonds) or 20 rescuers (magenta squares).

The value of the optimal probing depth increased for smaller search areas, since it takes less time to search the whole area, while the optimal probing depth decreased for larger search areas. The optimal probing depths for maximizing the survival chance of a buried subject as a function of avalanche debris size is shown in Fig 6.

The median size of the avalanche deposit for the dataset shown in Fig 1B is 7200 m^2^. However, it is rare that a buried subject is only found after the entire deposit has been probed. Entrance tracks of the caught subjects, terrain shape, direction of flow, potential anchoring points, witnessed "last seen points" as well as visual clues on the surface allow to determine the "most likely burial areas", thus in many cases strongly reducing the total area which needs to be probed.

Hence, the scenario including five rescuers and 5000 m^2^ surface is typical in companion rescue within the first 20 to 30 min after an accident. Due to restrictions of resources, the companions will be forced to focus on a strongly limited, most likely burial area. Once organized rescue arrives on scene and buried subjects are still missing, the initial search area can be extended as the availability of resources strongly increases. A probing depth larger than 2.50 m might also be considered, but only once organized rescue with professional, long probes has arrived on scene.

### Optimal resuscitation time

**Fig 7:**
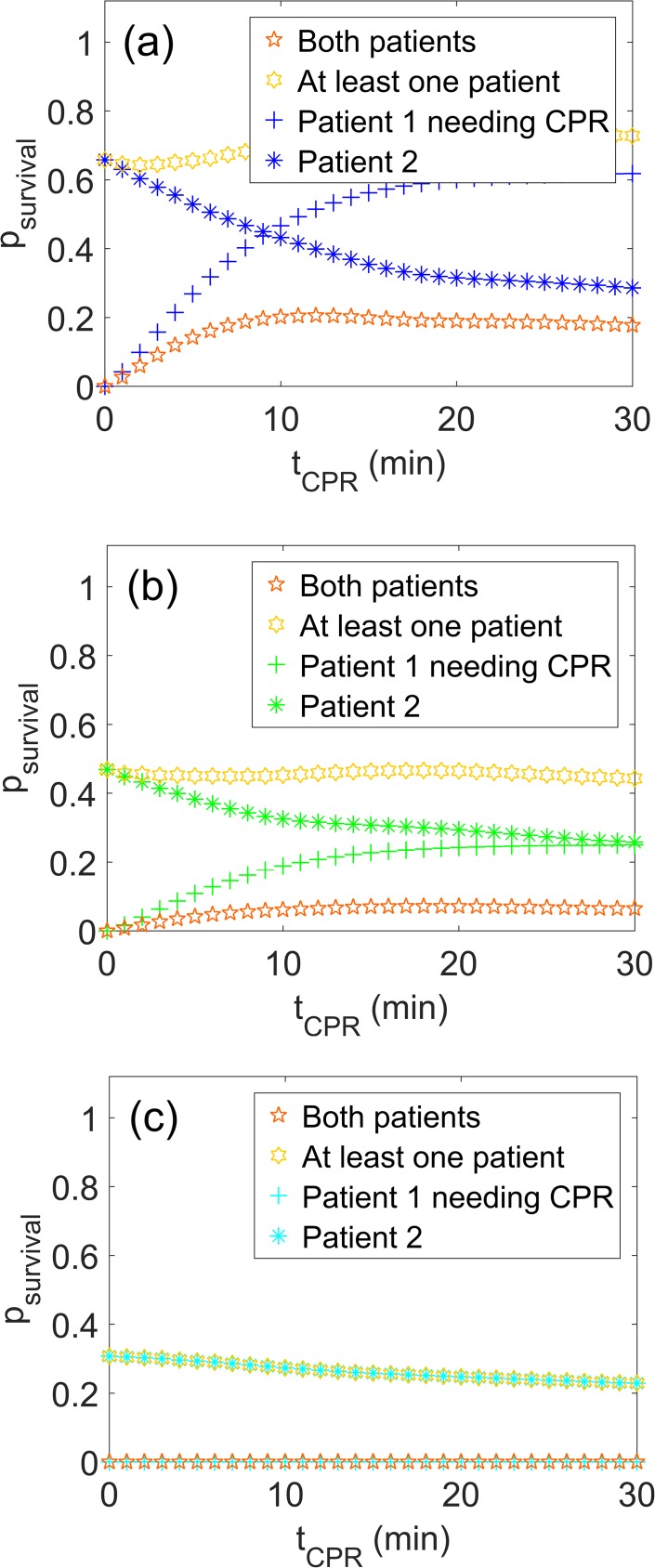
Survival curves. Single survival curves (blue/green/cyan crosses and stars) for patient 1 and patient 2 over *t*_CPR_, the time used to perform CPR on patient 1. The assumed burial time of patient 1 was (a) 12 min, (b) 20 min and (c) 35 min. Also shown is the probability of both patients surviving (open orange stars) as well as the probability that at least one of the patients survives (yellow hexagrams).

The single survival curves for patient 1 needing CPR and patient 2 waiting for excavation are shown in Fig 7 as a function of *t*_CPR_, the time used to perform CPR on patient 1. In addition, the probability, that both patients survive, as well as the probability, that at least one of the patients survives, are shown (*p*_1_∙*p*_1_: open orange stars and 1 − [(1 − *p*_1_) ∙ (1 − *p*_2_)]: yellow hexagrams). For a starting time of 12 min (Fig 7A), the survival chances of both patient 1 and patient 2 are still fairly high, with an average probability of survival of 0.47 and 0.40 for patient 1 and 2, respectively. While the survival chances of patient 1 increase with increasing *t*_CPR_, the survival chances of patient 2 decrease, since increasing *t*_CPR_ means increasing burial time for him or her. The probability of both patients surviving are highest for a *t*_CPR_ of 12 min with a value of 0.2, i.e. 20%. This means that even for the short initial burial time of 12 min, the probability that at least one patient dies is about 80%. The probability that at least one patient survives initially increases with increasing *t*_CPR_, to reach a constant value of 72% between 17 and 30 min.

The probabilities of survival depend strongly on the time it takes to excavate patient 1 and start performing CPR. If this time is assumed to be 20 min (Fig 7B), the pattern of the survival curves is still similar as for an initial burial time of 12 min, but the maxima are lower. The average probabilities of survival are 0.19 and 0.32 for patient 1 and patient 2, respectively, and the maximum probability of both surviving is 7% at *t*_CPR_ of 16 minutes. The maximum probability of at least one patient surviving again reaches a constant value at a *t*_CPR_ between 17 and 30 min but has dropped to 47%.

In the case of a relatively long initial burial time–i.e. the burial time of patient 1 before CPR starts–of 35 min, the survival chances of patient 1 reduce to zero [18]. The chances of patient 2 increase with time, starting at 31% (Fig 7C).

**Fig 8:**
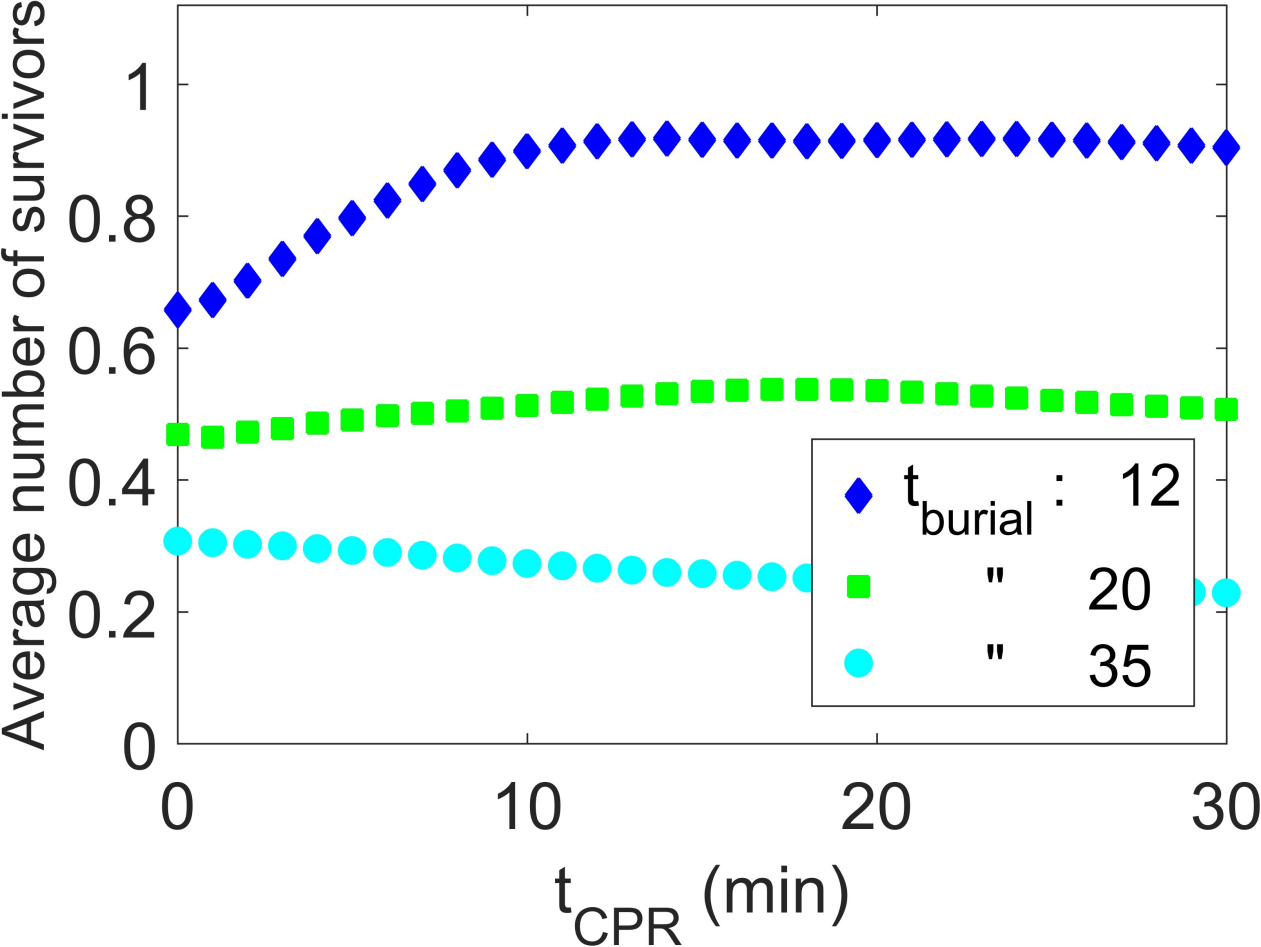
Number of survivors. Expected number of survivors over resuscitation time *t*_CPR_ for initial burial times for patient 1 of 12, 20, and 35 min.

In the sense of the “greatest good for the greatest number” we are interested in maximizing the number of survivors. The expected number of survivors as a function of resuscitation time *t*_CPR_ for different initial burial times of patient 1 is shown in Fig 8.

The maximum expected number of survivors is achieved for a CPR time of 11 min, for an initial burial time of patient 1 of 12 minutes, as can be seen from Fig 8. If it takes longer (20 min) to excavate patient 1 the expected number of survivors stays at roughly 0.5 as any gain in survival chances due to CPR performed on patient 1 is compensated by the drop of survival chances for patient 2 who is still buried. In the case of long initial burial times of patient 1, i.e. 35 min or longer, the expected number of survivor purely reflects the survival probability of patient 2 and is consequently decreasing with increasing *t*_CPR_.

Obviously, our simulations results depend on the input data. This is particularly true for the data on the probability of survival of patient 1. The data from Reynolds *et al*. [15] consisted of patients of the age group 56±15 years, 40% of which already had a pacemaker implanted. The cardiac arrest of the patients occurred spontaneously and was not triggered by hypoxia, as it happens commonly within the first 35 minutes in an avalanche. Accordingly, the patient group studied by Reynolds *et al*. [15] might not be an ideal representation of the average avalanche victim. However, we are not aware of any better representative data for our case. For typical avalanche victims, we assume that the curves in Fig 4 might increase even steeper. The steeper the curves in Fig 4 increase, i.e. the probability of survival increases with increasing duration of CPR, the shorter is the time for which the expected number of survivors is maximal. Moreover, the data presented by Reynolds *et al*. [15] was of normothermic patients, which is similar to avalanche burial within the first hour. Only for burial durations longer than 60 minutes, subjects may be hypothermic (<35°C) ([19], 20]). For the sake of simplicity, we have explicitly not considered any cooling. However, even assuming the fastest cooling ever recorded in an avalanche [20] our findings would not change substantially. The parameter *a* which quantifies the proportion of survivors as a function of the burial time of patient 1, i.e. the success rate of CPR was calculated from the data presented by Moroder *et al*. [18]. As the data are sparse, the values present a rough estimate only. The lower the success rate is the less pronounced is the maximum for the expected number of survivors–equivalent to proceeding immediately to patient 2. Finally, maximizing the survival chances for the whole scenario, i.e. following the “greatest good for the greatest number” principle, does not necessarily maximize the survival chances for each individual victim or the probability that at least one patient survives.

Despite the above-mentioned limitations it seems likely that in our simple triage scenario, given the simulation results, performing CPR on patient 1 for less than the 20 minutes recommended by Truhlář *et al*. [16] will overall maximize the sum of the survival chances of both patients. The steadily decreasing survival of patient 2 can hardly be compensated with performing an extended CPR on patient 1. Nevertheless, we do not mean to issue a new recommendation on the duration of CPR. Rather, our study should be considered as a proof of concept showing the potential of simulations in a rescue setting. Whereas our simulations cannot replace specific comprehensive data on avalanche rescue outcomes, they are useful to explore various competing effects for cases with very limited data.

Our study shows that certain data is required to optimize avalanche rescue procedures. To calculate the optimal probing depth we specifically require data on the probing speed as a function of probing depth, preferably also as a function of the number of rescuers. We assume that the probing speed in reality increases less than linearly with increasing number of rescuers as more rescuers need more time to line up after each set of probing strokes. In addition, for all simulations concerning avalanche rescue we need to know the digging speed as a function of burial depth and the number of rescuers. Finally, for the simulation including CPR, the survival probabilities of buried subjects who require CPR as a function of burial time are crucial.

## Conclusions

The application of numerical simulations based on the Monte Carlo principle allow finding optimal solutions in the realm of the "greatest good for the greatest number"–an approach previously not possible. For two optimization problems in avalanche rescue, probing depth and CPR duration in case of shortage of resources, we have exemplarily shown the potential of this simulation approach.

Our Monte Carlo simulations primarily provide exemplary results as we had to make several assumptions given that the underlying data are sparse or do not even exist. Nevertheless, we suggest to consider the optimized suggested values for probing depth in probe line searches and CPR duration while revisiting current search and rescue strategies as well as triage decision support systems. In particular, our simulations suggest that for the second triage scenario (i.e. resuscitation in case of an additional buried subject) a CPR duration shorter than the 20 min as recommended by Truhlář *et al*. [16] might help to maximize the expected number of survivors in the triage scenario presented. Whereas our Monte Carlo simulations cannot replace specific comprehensive data on avalanche rescue outcomes, they are useful to explore various competing effects for cases with very limited data. Hence, we consider our study as a first step demonstrating the usefulness of the simulation approach for optimization problems in avalanche rescue.

In the future, we plan to extend our simulations, and in particular gather more medical data on avalanche victims to improve the underlying assumptions, e.g. on the success rate of CPR efforts.

## Supporting information

S1 Metadata Avalanche data. Avalanche ID, burial depth (in cm) and avalanche deposit size (in m2) for fully buried subjects by avalanches in Switzerland recorded between 1973–1974 to 2012–2013 in the SLF avalanche database (1555 cases in total). Out of the 1555 cases either only the burial depth (1490 cases) or only the deposit size (541 cases) is known. For 477 cases both the burial depth and the deposit area were recorded.
(PDF)

S1 Table Avalanche data. Avalanche ID, avalanche burial depth, and avalanche deposit area.
(CSV)
