# Supplementary material for: A concept for optimizing avalanche rescue strategies using a Monte Carlo simulation approach
Source: PLoS One. 2017 May 3;12(5):e0175877. doi: 10.1371/journal.pone.0175877 (PMC5414947; doi:10.1371/journal.pone.0175877)
Supplement: S1 Metadata — Avalanche ID, burial depth (in cm) and avalanche deposit size (in m2) for fully buried subjects by avalanches in Switzerland recorded between 1973–1974 to 2012–2013 in the SLF avalanche database (1555 cases in total). Out of the 1555 cases either only the burial depth (1490 cases) or only the deposit size (541 cases) is known. For 477 cases both the burial depth and the deposit area were recorded. (PDF) [file pone.0175877.s001.pdf]

Avalanche data

Avalanche ID, burial depth (in cm) and avalanche deposit size (in m^2) for fully buried subjects by avalanches in Switzerland recorded between 1973-1974 to 2012-2013 in the SLF avalanche database (1555 cases in total).

Out of the 1555 cases either only the burial depth (1490 cases) or only the deposit size (541 cases) is known. For 477 cases both the burial depth and the deposit area were recorded.
